# Generalized Analysis of Molecular Variance

**DOI:** 10.1371/journal.pgen.0030051

**Published:** 2007-04-06

**Authors:** Caroline M Nievergelt, Ondrej Libiger, Nicholas J Schork

**Affiliations:** 1 Department of Psychiatry, University of California at San Diego, La Jolla, California, United States of America; 2 Department of Family and Preventive Medicine, University of California at San Diego, La Jolla, California, United States of America; 3 Rebecca and John Moores UCSD Cancer Center, University of California at San Diego, La Jolla, California, United States of America; 4 The Center for Human Genetics and Genomics, University of California at San Diego, La Jolla, California, United States of America; 5 The Stein Institute for Research on Aging, University of California at San Diego, La Jolla, California, United States of America; 6 Scripps Genomic Medicine and Department of Molecular and Experimental Medicine, The Scripps Research Institute, La Jolla, California, United States of America; University of Alabama at Birmingham, United States of America

## Abstract

Many studies in the fields of genetic epidemiology and applied population genetics are predicated on, or require, an assessment of the genetic background diversity of the individuals chosen for study. A number of strategies have been developed for assessing genetic background diversity. These strategies typically focus on genotype data collected on the individuals in the study, based on a panel of DNA markers. However, many of these strategies are either rooted in cluster analysis techniques, and hence suffer from problems inherent to the assignment of the biological and statistical meaning to resulting clusters, or have formulations that do not permit easy and intuitive extensions. We describe a very general approach to the problem of assessing genetic background diversity that extends the analysis of molecular variance (AMOVA) strategy introduced by Excoffier and colleagues some time ago. As in the original AMOVA strategy, the proposed approach, termed generalized AMOVA (GAMOVA), requires a genetic similarity matrix constructed from the allelic profiles of individuals under study and/or allele frequency summaries of the populations from which the individuals have been sampled. The proposed strategy can be used to either estimate the fraction of genetic variation explained by grouping factors such as country of origin, race, or ethnicity, or to quantify the strength of the relationship of the observed genetic background variation to quantitative measures collected on the subjects, such as blood pressure levels or anthropometric measures. Since the formulation of our test statistic is rooted in multivariate linear models, sets of variables can be related to genetic background in multiple regression-like contexts. GAMOVA can also be used to complement graphical representations of genetic diversity such as tree diagrams (dendrograms) or heatmaps. We examine features, advantages, and power of the proposed procedure and showcase its flexibility by using it to analyze a wide variety of published data sets, including data from the Human Genome Diversity Project, classical anthropometry data collected by Howells, and the International HapMap Project.

## Author Summary

Humans exhibit great genetic diversity. Understanding the factors that contribute to and sustain this diversity is an important research area. Not only can such understanding shed light on human origins, but it can also assist in the discovery of genes and genetic factors that contribute to debilitating diseases. Statistical analysis methods that can facilitate the identification of factors contributing to or associated with human genetic diversity are growing in number as new high-throughput molecular genetic assays and technologies are developed. We consider the use of an analysis method termed generalized analysis of molecular variance (GAMOVA), which builds off of previously proposed analysis methods for testing hypotheses about the factors associated with genetic background diversity. We apply the method in a wide variety of settings and show that it is both flexible and powerful. GAMOVA has great potential to assist in population-based human genetic studies, as it can be used to address questions such as: Is a sample of affected cases and unaffected controls from a homogeneous population, or is there evidence of heterogeneity that could affect the results of an association study? Is there reason to believe that the ancestry of a set of individuals influences the traits that they have?

## Introduction

Genetic and genetic epidemiologic studies involving large numbers of individuals and/or populations are being pursued more and more often as a result of the development of high-throughput genotyping technologies and the creation of genotype data repositories such as the dbSNP (http://www.ncbi.nlm.nih.gov/SNP) and the International HapMap Project databases (http://www.hapmap.org). Many of these studies are concerned with the identification and characterization of the relationships of the populations and/or subsets of individuals in those populations on the basis of their genomic profiles or “genetic backgrounds” (i.e., whether or not these populations/individuals carry the same sets of genetic variations [1–8]). In addition, genetic epidemiologic studies are often conducted to identify relationships between specific sets of genetic variations possessed by individuals and phenotypic endpoints they might have, such as a disease. The collection of variations that an individual possesses that contribute, e.g., to his or her disease susceptibility, may vary from population to population (e.g., as defined geographically, ethnically, racially, or linguistically). This may be due to the underlying heterogeneity of disease pathogenesis, the origins of the variations both in terms of time and place, and the frequency with which those variations are transmitted across populations (e.g., via migration patterns, interpopulation matings, etc.). Thus, the genetic background of an individual—at least with respect to relevant disease-contributing variations—is as crucial in these types of investigations as it is in other types of population genetic studies. In addition, it has been shown that, due to phenomena such as varying degrees of admixture and/or cryptic relatedness in the study population, ignoring genetic background in epidemiologic studies testing associations between particular genetic variations and a phenotype can result in false positive and false negative results [9–19], which underscores the importance of genetic background analysis even in very simple genetic association studies.

Many innovative analytical methods have been developed recently to assess and accommodate genetic background heterogeneity [20–37]. The vast majority of these methods involve some form of cluster analysis, although some more recent methods do not (e.g., [29,32]). For example, hierarchical clustering strategies can be used to assess genetic background clustering, and, like other cluster analysis methods, require the construction of a measure of the similarity or dissimilarity (genetic distance) between all pairs of the *N* individual genomes or population allele frequency profiles (e.g., between-group variation, *F*_ST_) comprising a sample. The resulting *N* × *N* similarity or distance matrix is then explored statistically to identify clusters of individuals or populations that exhibit greater or lesser similarity. Problems inherent to this approach involve the choice of a similarity metric, deciding which cluster method is most appropriate (e.g., single linkage, complete linkage, etc.), the determination of the optimal number of clusters representing the data, and the biological meaning of the clusters.

With respect to the choice of a similarity metric for cluster analysis, the simplest marker-based method for the assessment of genetic similarity between two individuals is to calculate the fraction of alleles shared identical by state (IBS) by those individuals over all the loci for which the individuals have been genotyped. If *N* individuals have been genotyped, then all *N* × *N* pairs of individuals can be assessed in this way. In addition to providing a foundation for some cluster analysis methods, graphical displays of the similarity matrix can be produced that allow visual assessment of the potential that subgroups of individuals with similar genetic backgrounds exist in the data. This approach has been used widely, and is often referred to, when presented in graphical form as a dendrogram, or as an allele-sharing “tree of individuals” (e.g., [7,38–40]). One problem, however, with the simple IBS sharing measure of genetic background similarity is that it does not account for allele frequencies. Consider, for example, two individuals who share rare alleles. These individuals are more likely to have arisen from the same (unique) population in which those alleles arose. In this situation one may want to consider “weighting” allele sharing at each locus by the frequency of the shared (or unshared) alleles. Pairwise measures of genetic similarity that accommodate allele frequencies have been put forward and are used often in ecological and nonhuman population genetics analysis settings (e.g., [41–45]).

Cluster analysis approaches can be extended by making more explicit and rigorous assumptions about the ancestral populations from which the individuals in a sample arose. Thus, specific ancestry informative markers (AIMs), which show large frequency differences between ancestral populations, can be used to quantify the degree of admixture among individuals in a sample [18,46–49]. When an individual genotyped on such markers possesses variations that are more frequent in one of the chosen ancestral populations, then that individual's ancestral relationship to this population can be inferred. Obviously, one needs to have identified the appropriate AIMs in advance of such analyses and this requires assumptions about the ancestral populations contributing to the individual genetic backgrounds reflected in a sample.

In the following we describe a flexible alternative to cluster analysis–based methods for the statistical assessment of genetic background similarities among populations or individuals. The proposed method does not necessarily rely on AIMs, but does require genotype information on at least a few hundred (possibly less when including AIMs) genetic markers (null loci) such as microsatellites, single nucleotide polymorphisms (SNPs), and/or insertion–deletion polymorphisms. Although one can use markers that are not completely independent in the sense that they have alleles in linkage disequilibrium, this practice may require the use of a greater number of markers to make up for the lack of independence of the markers. Null loci can include genotype data available from, e.g., a previous genome-wide association or linkage studies involving the subjects or populations of interest, and could thus allow for a retrospective analysis of sample genetic background structure without additional genotyping. As in cluster analysis, the proposed method involves the construction of a genetic similarity matrix. However, it does not require cluster analysis to test hypotheses about the relationships of the individuals or populations in a sample. Rather, the method assumes that interest lies in testing the relationship between a particular grouping factor (e.g., race, country of origin, cohort, or geographical locale) or quantitative measure (such as age, cholesterol level, or weight) and variations in the genetic similarities of the individuals or populations collected. Therefore, it does not require the determination of the optimal number of clusters or, e.g., principal components, representing the data.

The proposed method is similar to the analysis of molecular variance (AMOVA) method introduced by Excoffier and colleagues, but is more flexible and provides a much more intuitive and generalizable derivation of relevant test statistics [50]. The description of the AMOVA procedure provided by Excoffier et al.[50] includes relevant sum-of-squares calculations to formulate analysis of variance (ANOVA)-like hypothesis-oriented test statistics that consider differences between groups of individuals or populations with respect to genetic background. As described in the Methods section, the proposed approach builds off an analysis method we have termed multivariate distance matrix regression analysis and can be used to test hypotheses about not only categorical or grouping factors and genetic background, but quantitative traits as well [51,52]. In addition, the formulation of the proposed test statistics can be adapted for use in multiple regression-like test settings, so that the relationships of multiple factors to genetic background can be explored. As a result of the connections between the proposed approach and the AMOVA approach of Excoffier et al. [50], we have labeled the proposed approach generalized molecular analysis of variance (GAMOVA).

In addition to the AMOVA procedure, the proposed GAMOVA procedure also has some similarities to the Mantel-based test statistic approach reviewed and extended by Smouse et al. [53]. The Mantel test is used to test the relationship between the entries or cells in two (or more) distance/similarity matrices. Thus, one could have a genetic background similarity matrix computed from different populations whose relationship to, e.g., a geographic distance matrix computed for the populations is of interest. The proposed GAMOVA procedure considers the relationship between the *N* × *N* entries (or cells; where *N* is the number of individuals or populations being studied) in a genetic background distance matrix and information, represented as *N*-dimensional vectors, on the *N* individuals or populations whose genetic background distances are reflected in the matrix.

Below we apply the GAMOVA procedure to three data sets available in the public domain to address some prevailing questions: (1) an analysis of the Foundation Jean Dausset-Centre d'Etude du Polymorphisme Humain (CEPH)–Human Genome Diversity Project (HGDP) Cell Line Panel, (2) an analysis of the morphological data made available by Howells [54,55] on human craniometric characters, and (3) an analysis of the International HapMap Project data addressing questions about the similarity of the individual chromosomes possessed by the subjects genotyped as part of the project. In addition, we also consider aspects of the power of the GAMOVA procedure via simulation studies.

## Results

### Analysis of the CEPH-HGDP Cell Line Panel Dataset

We considered the use of the proposed GAMOVA analysis to analyze the CEPH-HGDP Cell Line Panel data [56] in a number of ways. We constructed several distance matrices over 1,040 subjects collected from 51 worldwide populations based on: (1) individual IBS allele sharing and (2) Lynch-Ritland (LR) frequency weighted allele-sharing distance, and (3) the standard between-population genetic distance measure *F*_ST_ (see Methods). We then considered the relationship between additional information collected on those individuals (and/or populations) and variation in the similarity among the individuals and populations using the proposed GAMOVA procedure. The additional information included, for each individual, which of the 51 populations or ethnic groups they were from, the geographic location of that population (i.e., one of the five or seven global world regions associated with populations), and its distance from Addis Ababa in Africa [8]. In addition to the analyses based on individuals, geographic location and distance from Addis Ababa were also considered in analyses involving the 51 populations as a whole. By considering the distance of each population from Addis Ababa we could address hypotheses about global historical migration patterns and the impact these migration patterns have on genomic diversity, as has been recently pursued through the use of different statistical methods [6,57].

**Figure 1:**
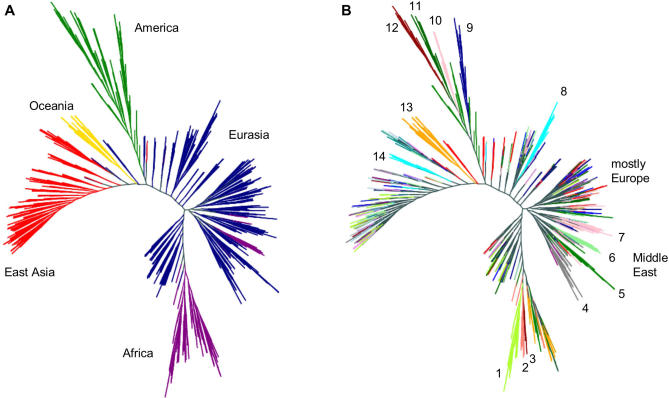
Neighbor-Joining Trees Depicting the Genetic Relationships of 1,040 Individuals from 51 World Populations Collected by the CEPH-HGDP (A) Individuals are color coded according to which of five major geographic regions of the globe they are collected from. (B) Individuals are color coded according to which of the 51 populations they are associated with (1: Biaka Pygmy, 2: San, 3: Mbuti Pygmy, 4: Druze; 5: Bedouin, 6: Mozabite, 7: Palestinian, 8: Kalash, 9: Pima, 10: Columbian, 11: Karitiana, 12: Surui, 13: New Guinea, 14: Yakut).

To visually assess the potential for genetic background clustering we first constructed neighbor-joining trees based on the IBS distance matrix of the CEPH-HGDP individuals. We color-coded each branch (representing an individual) based on: (1) which of 5 major geographic regions (Figure 1A, left panel) and (2) which of 51 populations an individual was from (Figure 1B, right panel). Figure 1 shows a fairly dramatic clustering of the individuals that is roughly consistent with the population of origin for each individual. Note that, as observed by Rosenberg et al. [1], the Mozabite (the population labeled with a “6”), a Berber ethnic group living in the Sahara in Northern Africa, clusters with Middle Eastern populations (assigned labels “4,” “5,” and “7”).

We then considered two analyses designed to assess how much of the genetic background variation exhibited by the CEPH-HGDP individuals and ethnic groups could be explained by the world regions each individual or population was associated with, as well as the distance of that world region from Addis Ababa, using the GAMOVA procedure. We created simple 0–1 indicator variables that reflected which world region an individual or population was associated with and used these indicator variables as independent or predictor variables in the GAMOVA regression procedure (see the Methods section for details) along with distance from Addis Ababa as a continuous variable. Table 1 provides the results assuming either a seven-world region breakdown (Table 1; East Asia, Africa, Oceania, Central and South Asia, America, the Middle East, and Europe) or a five-world region breakdown (Table 1; Eurasia, East Asia, Oceania, America, and Africa) as defined previously [1]. We also compare GAMOVA regression models that did not consider (Table 1) distance from Addis Ababa as a predictor to contrast the results with the findings of models that included it (Table 1).

**Table 1:**
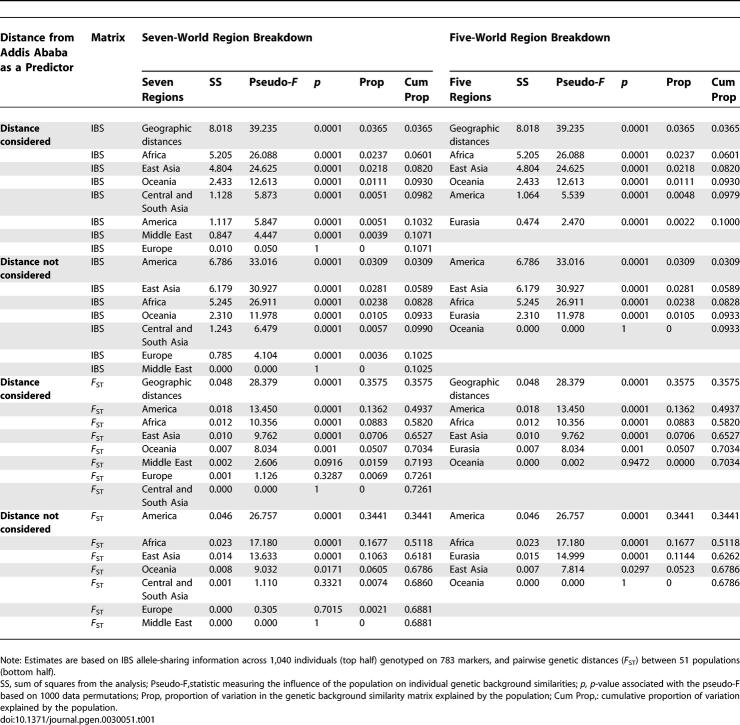
GAMOVA Analysis Estimates of the Poportion of Variation in Genetic Background Similarity Explained by Seven or Five World Regions, Respectively, Including and Excluding Geographic Dstances (between Each Population and Addis Ababa, See Text)

The top half‘ of Table 1 reflects the analysis of the IBS allele sharing among individuals and suggests that approximately 9%–11% of the variation in the similarity of individual genetic backgrounds can be explained by world region either in conjunction with the distance of that world region from Addis Ababa or not. Approximately 68%–72% of the variation in genetic background similarity of the populations as a whole, assuming the *F*_ST_ measure of genetic distance, could be explained by world region and distance of those world regions from Addis Ababa (Table 1; bottom half). This clearly reflects the greater diversity among individual genomes within a population than allele frequency differences between populations as a whole.

It is also interesting to note that, as found by others [6,57], the distance from Addis Ababa is the strongest predictor of genetic background similarity among the individuals and populations, but the world regions explain variation in genetic background similarity over and above this measure, suggesting that diversity among individuals within populations situated within the same world region is not completely captured by their distance from Addis Ababa. Also of note is the strength of the contributions of the various world regions to variation in genetic background similarity, which reflect factors such as the populations' individual demographic histories and selective environmental pressures. For example, Africa is the strongest contributor to individual genetic background similarity after accounting for each world region's distance from Addis Ababa (Distance considered/IBS Matrix portion of Table 1), which is consistent with the deep genetic structure of this continent [58]. On the other hand, the strongest contributor to pairwise population distances (*F*_ST_) after accounting for geographic distance from Addis Ababa (Distance considered/*F*_ST_ portion of Table 1) was found to be America, consistent with findings by Ramachandran et al. [58].

We also considered analyses that took into consideration all the populations studied, assuming both the IBS allele-sharing measure of genetic background similarity and the LR allele frequency-weighted measure (Table 2). Overall, the individual populations that the study subjects were from could explain approximately 16%–19% of the variation in genetic background similarity exhibited by the individuals in the CEPH-HGDP database. Interestingly, the analyses using the IBS and LR measures did not agree perfectly—although they are similar—suggesting that allele frequency weighting can make a difference in assessing individual genetic background similarity. In addition, our GAMOVA analysis suggests that individuals from three populations in the Americas (the Surui, the Karitiana, and the Pima) have the most divergent genomes from the other individuals' genomes, which has been observed by others as well (e.g., [1,58]).

**Table 2:**
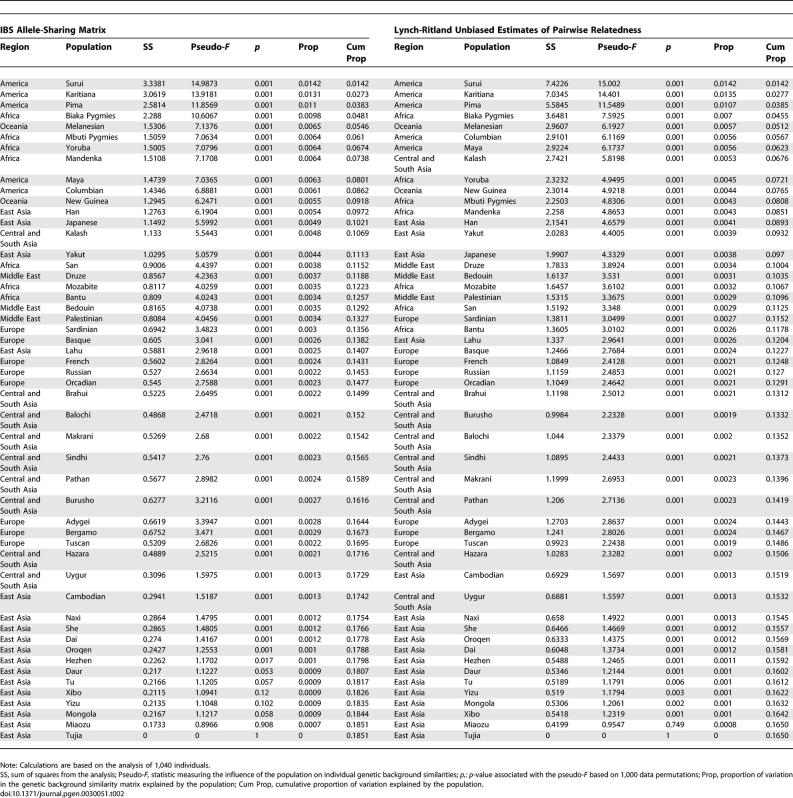
GAMOVA Analysis Estimates of the Proportion of Variation in CEPH-HGDP Individual Genetic Background Similarity Explained by the 51 Populations from Which the Subjects Were Collected on the Basis of IBS Allele-Sharing Information (Left Half) and an Allele Frequency Weighted Measure LR (Right Half)

### Analysis of the Craniometric Data Collected by Howells

We also considered analyses involving morphological data made available by Howells [54,55] on human craniometric characters collected on individuals from ten worldwide populations. We computed the median of each of 43 craniometric measures for males and females separately from each of these populations. We combined the data with the genetic data on the CEPH-HGDP subjects by geographically matching the countries and regions represented in the CEPH-HGDP with those for which we had craniometric data in a fashion identical to the one outlined by Roseman [59]. The median values for each of the 43 craniometric measures were then considered as a regressor or covariate in a GAMOVA analysis of the genetic distance matrix computed for the ten corresponding CEPH-HGDP populations. The goal was to test associations between craniometric features of the people within the populations and genetic background similarities those people might have with people in other populations. We want to emphasize that many of the craniometric measures are correlated so that associations between any one of these measures and genetic background suggest that other measures may also be associated with genetic background, just not necessarily independently of the others.

Table 3 describes the results of the analyses for males and females. The cranial feature most strongly associated with genetic background similarity is the nasion-bregma subtense (FRS), which “explains” ∼54% and ∼49% of the variation in genetic background similarity for males and females, respectively. Other measures, such as glabella projection, minimum cranial breadth and basion-prosthion length for males, and brasion-prosthion length and dacryon subtense for females, were found to be also associated with variation in population genetic profile similarity over and above the FRS. The multicollinearity among the 43 measures precluded fitting a GAMOVA model with all 43 measures as predictors, so only those measures that had associations with genetic background similarity that were independent of the others were considered in Table 3 (i.e., as in standard multiple regression contexts). A strong association between the frontal bone curvature FRS with genetic background has also been reported by Roseman and Weaver using a principal components analysis [60]. As found by others (e.g., [61]), our analysis suggests that certain morphological features, namely craniometric features, segregate with genetic background across different global populations much like, e.g., skin color [62].

**Table 3:**
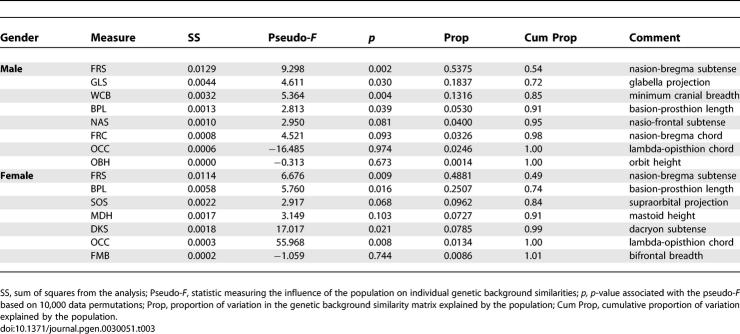
GAMOVA Analysis Investigating the Relationship between Craniometric Measures Collected by Howells and Genetic Background

### Analysis of the HapMap Dataset

We next considered the application of the GAMOVA procedure to the analysis of the large-scale genotyping effort associated with the International HapMap Project (http://www.hapmap.org; [63]). The data consist of genotypes at over a million SNP loci on 209 individuals associated with four different population groups (Northern European, West African, Japanese, and Han Chinese). Computational methods were used to “phase” individuals based on the genotype data (i.e., probabilistically assign unique chromosome pairs to each individual based on linkage disequilibrium patterns) by the HapMap investigators; see the description of the phasing procedures at the International HapMap Project Web site. We undertook an analysis investigating the fraction of genetic similarity explained by the four population subgroups on a per-chromosome basis using a simple IBS measure of genotype similarity for the 209 individuals, as well as individual chromosomal similarity based on the 209 × 2 = 418 chromosomes obtained from the phase-resolved data. The goal of this analysis was to determine how much of the similarity or distances between the multilocus diploid genomes, on a per-chromosome basis, could be explained by the population groups associated with the HapMap individuals. We also wanted to determine how much of the similarity or distances between the individual chromosomes (with each person contributing two to the total pool of 209 × 2 = 418) could be explained by the population groups associated with the Hapmap individuals.

Table 4 describes the results and suggests that roughly 20%–22% of the individual chromosomal similarity can be explained by the populations associated with each chromosome (i.e., assigned haplotypes) (left half of Table 4) and roughly 28%–30% of the individual chromosomal similarity based on each individual's diploid genotype can be explained by the population origins of the subjects. We note that the percentages are consistent across the chromosomes, as one might expect. In addition, the Yoruban population has the most divergent chromosomes, followed by the Northern Europeans. The distinction between the Han Chinese and Japanese chromosomes, although significant, is much weaker, as expected, since the residual variation after accounting for African and European background effects is very small. In addition, whereas the effect of Chinese origin was more significant on individual chromosome similarity, the effect of Japanese origin was more significant on genotyping similarity.

**Table 4:**
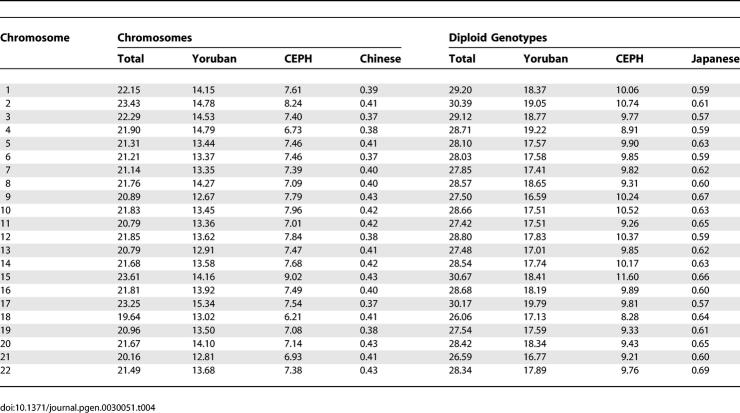
Percentage of the Variation in the Dissimilarity of Individual Chromosomes or Diploid Genotypes Explained as a Function of the Population Designations of the 209 Subjects Genotyped as Part of the HapMap Project

### Power Estimation

**Figure 2:**
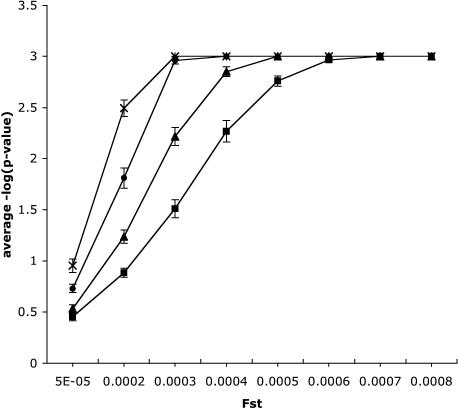
Relationship between the Genetic Differentiation among Two Populations as Measured by Wright's *F*_ST_ and the Average (±S.E.M.) Power of the GAMOVA Procedure to Detect that Differentiation Results are based on 1,000 simulation studies involving four sets of two equally sized populations, each generated according to varying genetic differentiation. Known group membership was used as predictor in the GAMOVA analysis. For a constant data size (number of markers × number of subjects), genetic differentiation can be detected at lower *F*_ST_ values in larger populations with fewer markers compared to smaller populations with more markers (squares: 32 individuals, 32768 markers; triangles: 64 individuals, 16384 markers; circles: 128 individuals, 8192 markers; stars: 256 individuals, 4096 markers).

We also considered the power of the proposed GAMOVA procedure to detect varying degrees of differentiation between two populations using simulated data. We chose simulation settings that were consistent with those recently described by Patterson et al. [64]. We simulated four different settings/datasets with two populations each, whose pairwise genetic distances ranged from *F*_ST_ = 0 to *F*_ST_ = 0.01 (see Methods). We performed a GAMOVA analysis on these data with known group membership taken as a predictor variable. These analyses were repeated for a total of 1,000 simulations in each setting. Results were binned in groups having different *F*_ST_ statistics calculated for each data set (i.e., knowing the assumed *F*_ST_ used to generate the data may differ from the *F*_ST_ calculated from the simulated sample). Figure 2 shows the relationship of *F*_ST_ between the two populations to power of GAMOVA to detect that level of differentiation at a type-I error rate of 0.05. In general, GAMOVA shows excellent power at very low *F*_ST_ values around 0.0002, which is in the range of the least differentiated human populations described in literature (e.g., for different geographic regions of Iceland, a homogenous genetic isolate [14]). As noted by Patterson et al. [64], we found that at a fixed data size (*D* = number of markers × number of subjects), genetic differentiation is easier to detect for larger sample sizes, even though a smaller number of markers is used, than for smaller sample sizes using a larger number of markers.

## Discussion

As DNA sequencing and genotyping costs decrease, a greater number of population scientists, geneticists, clinical researchers, and epidemiologists will seek to identify and characterize genetic variations that underlie phenotypic variations as well as the biological relationships among individuals. Flexible analysis tools that can be used to test appropriate hypotheses will thus be needed for these investigations. We have proposed an analysis procedure, GAMOVA, that not only extends an analysis of variance approach that is used widely [50] for assessing relationships between genetic variations, phenotypic variations, and the population origins of individuals, but also complements widely used cluster analysis approaches for these purposes [20,23]. Specifically, the proposed GAMOVA approach can be used to test hypotheses about the relationship between variables collected on individuals or populations (such as particular phenotypes or population-level migration patterns) and variation in the genetic similarity or distance of those individuals or populations as characterized by individual genotype or allele frequency data.

Our applications of the proposed GAMOVA procedure suggest that it can be used to address a number of population genetic questions concerning the relationships of individuals at the DNA sequence level; e.g., it can be used to directly quantify the degree to which certain factors, such as race, self-reported ethnicity, admixture, migration patterns, and anecdotally derived connections between individuals and populations, are associated with the genetic similarity of individuals and populations. The exploration of such relationships has been the hallmark of applied population genetics research for decades [65–68]. However, one particularly important area of application for the proposed procedure is in the area of genetic epidemiology, and genetic association studies in particular, for at least two reasons. First, it is well known that the polygenic and/or multifactorial nature of many traits and diseases can influence the identification of the individual loci contributing to the expression of those traits and diseases if not accounted for appropriately [69,70]. Second, it is also well known that population stratification or genetically distinct subdivisions within a population sampled for an association study can lead to both false positive and false negative results if ignored [12–16,18,69]. In these two contexts, the proposed GAMOVA approach can be used to test hypotheses about the relationship between a phenotype of interest and genetic background similarity among the subjects to be used in an association study (provided that they have been genotyped on an appropriate set of markers to assess genetic background [28,71,72]). If an association is found, then steps can be taken to accommodate the influence of genetic background on the trait or disease in question as described by many researchers. The steps that can be taken to control for genetic background heterogeneity within the context of the GAMOVA analysis could involve identifying the leading eigenvalues of the distance/similarity matrix and using the corresponding eigenvectors as regressor variables or covariates in an appropriate linear model relating the specific genetic variation in question to the phenotype of interest [29,64].

Properties of the GAMOVA procedure, i.e., its robustness, power, level accuracy, etc., have been studied in some very general contexts, such as those involving genetic association analyses, gene expression analyses, and DNA sequence–based association studies [51,52], as well as in the simulation studies presented here. For population genetic analyses, our simulations suggest that the GAMOVA procedure is sensitive enough to detect very low levels of population structure in epidemiological samples. In addition, the use of permutation tests provides a very robust method for testing hypotheses demonstrating that the procedure is powerful in many different settings. In addition, virtually all of these studies document the flexibility of the method.

In addition to the applications showcased here, as well as those outlined by Wessel and Schork [51] and Zapala and Schork [52], we have routinely made use of the GAMOVA analysis to test for, e.g., differences across studies due to laboratory effects or genotyping artifacts, genotyping quality shifts over time, and genetic background differences between subjects from an original and replication sample [73]. Finally, the GAMOVA procedure is also applicable to the identification of informative markers for specific cohorts or communities under study, since one can use the procedure to test the effect of each SNP on variation in a genetic background similarity matrix for informativeness without requiring knowledge about the ancestral history of the subjects under study.

There are, however, a few limitations inherent in the proposed GAMOVA approach that may provide fertile ground for further research. For example, the choice of a similarity or distance measure is crucial. Although the IBS and LR measures for individual genetic similarity and the *F*_ST_ and related measures for population-level genetic similarity (e.g., [6]) are the standards, it is unclear which of these measures are the most powerful to use in the GAMOVA procedure (or even other methods relying on distance measures besides GAMOVA). In this context the power of the proposed GAMOVA approach in different population analysis settings and locus effect scenarios deserves detailed attention. However, since the procedure is rooted in the derivation of traditional ANOVA, regression, and general linear models, many of the same intuitions and findings related to the power of these modeling procedures apply. For example, the proposed procedure assesses the question of how much of the variation in the similarity/dissimilarity exhibited by a group of individuals can be explained by another factor, which is analogous to questions concerning how much of the variation in a quantitative particular trait is explained by a certain factor in regression and ANOVA contexts.

A final concern with the proposed approach, which is an issue with all analysis methodologies that involve high-dimensional data types, involves missing genotype data. One can handle missing genotype data in a number of ways. First, one could restrict the construction of the similarity measure to only those individuals with complete data—which may result in a substantially reduced sample—or simply construct the measure with the data that are available on each pair of subjects. This latter approach will be problematic if a number of individuals are missing genotype data at the most heavily weighted (e.g., functional or informative) loci. Another approach to handling missing data would involve imputing or assigning individuals genotype data based on linkage disequilibrium information. This approach would only be as useful as the strength of the linkage disequilibrium between alleles at the loci with missing data and those without. The approach we took to handling missing data was to use whatever genotype information was available on the subjects for the similarity calculations.

Finally, we note that a web-based GAMOVA tool is available from the authors at http://polymorphism.scripps.edu/∼cabney/cgi-bin/mmr.cgi.

## Materials and Methods

### Computing a similarity matrix.

As noted, the proposed procedure requires the computation of a “distance” matrix that reflects the dissimilarity of the genetic backgrounds of the individuals or populations being analyzed. There are many possible measures that could be used to construct such a matrix, and we considered two methods for computing the similarity of individuals' genetic backgrounds based on genotype data collected on them. The resulting similarity measure can be translated into a distance or dissimilarity measure as described later. The first similarity measure is widely used and is based on simple IBS allele sharing [38] and can be calculated as the fraction of alleles shared identical by state for each pair of individuals in a sample over all the loci for which the individuals have been genotyped:

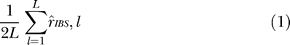
where **r̂*ibs* is the individual, locus-specific allele-sharing value and *L* = number of loci considered in the calculations.

The second similarity measure essentially considers weighting loci in the computation of IBS-based allele sharing by allele frequency and was introduced by Lynch and Ritland [44]. The LR regression-based method-of-moments estimator has been shown to have some desirable properties relative to other methods, especially in the case of populations consisting of individuals with a low degree of relatedness [45,74], and has been widely discussed in the population genetics and behavioral ecology literature (e.g., [75–77]). The LR estimator uses a regression approach to infer relationships (i.e., one individual of a pair serves as a “reference” individual and the probabilities of the locus-specific genotypes of the second individual are then conditioned on those of the reference individual). The LR coefficient of relatedness is:

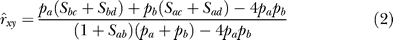
where *p_a_* and *p_b_* equal the frequencies of alleles *a* and *b* in the population. The reference individual is assumed to have alleles *a* and *b* (such that if this individual is homozygous, *S_ab_*=1, if heterozygous, *S_ab_* = 0), and the proband has alleles *c* and *d.* Multilocus estimates of genetic background similarity can be obtained by summing the single estimates, weighted by the inverse of their sampling variance:

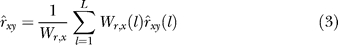
where

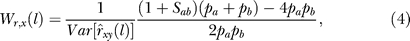
which is computed under the assumption that the two individuals in question are unrelated (i.e., have 0.0 relatedness).

The similarity matrices were transformed into a dissimilarity or “distance” matrix by subtracting the components of the matrix from 1.0 if the IBS measure is used, or subtracting them from 1.0 after each component in the matrix is divided by the theoretical or empirical maximum of the similarity measure to scale the entries to lie between 0 and 1.

### Multivariate distance matrix regression analysis.

Once one has computed a distance matrix it can be subjected to a regression analysis testing hypotheses regarding, e.g., whether or not variation in the level of similarity/dissimilarity exhibited by pairs of individuals reflected in that matrix can be explained by other features those individuals posses (e.g., whether they are from a particular ethnic group or a specific country). To describe the regression model, we assume that each of *N* individuals or study subjects has been genotyped at *L* unlinked polymorphic loci (bi- or multiallelic) and that *M* grouping or phenotypic variables have been collected on the *N* subjects. These grouping or phenotypic variables could include information about the country of origin (coded using dummy variables, such as a 1 assigned to individuals from a particular country, and 0 assigned to individuals from a different country), the continental origin of that country and its distance from Addis Ababa, and craniometric diversity data, as we have considered.

We note that the proposed regression procedure, which is an extension of the procedure described by McArdle and Anderson [78] and a general reformulation of the AMOVA procedure discussed by Excoffier et al. [50], does not require that the distance matrix used have metric properties. Let this distance matrix and its elements be denoted by *D = d_ij_ (i,j = 1,…,N),* for the *N* subjects. The possibility that *N ≪ L* will not pose problems in the proposed regression analysis setting. Let *X* be an *N* × *M* matrix harboring information on the *M* grouping or phenotypic variables, which will be modeled as predictor or regressor variables whose relationships to the values in the genomic similarity matrix are of interest. Compute the standard projection matrix, *H = X*(*X′X*)^−1^ *X′*, typically used to estimate coefficients relating the predictor variables to outcome variables in multiple regression contexts. Next, compute the matrix 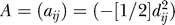 and center this matrix using the transformation discussed by Gower [79] and denote this matrix *G*:

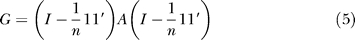



An *F*-statistic can be constructed to test the hypothesis that the *M* regressor variables have no relationship to variation in the genomic distance or dissimilarity of the *N* subjects reflected in the *N* × *N* distance/dissimilarity matrix as [78]:

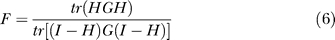



If the Euclidean distance is used to construct the distance matrix on a single quantitative variable (i.e., as in a univariate analysis of that variable) and appropriate numerator and denominator degrees of freedom are accommodated in the test statistics, the *F*-statistic above is equivalent to the standard ANOVA *F*-statistic [78]. The distributional properties of the *F*-statistic are complicated for alternative distance measures computed for more than one variable, especially if those variables are discrete, as in genotype data. However, permutation tests can then be used to assess statistical significance of the pseudo *F*-statistic [80,81]. The *M* regressor variables can be tested individually or in a step-wise manner. All matrix-based regression analyses we have performed in this paper used 10,000 permutations to calculate *p*-values, except for the analysis of the CEPH-HGDP data in Table 2, for which we used 1,000 permutations. In addition, one can calculate the percentage of variation in similarity/distances within the distance matrix explained by the regressor variables, *r* ^2^, through the formula:

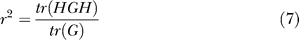



### Graphical display of similarity matrices.

Similarity matrices of the type we have described can be represented graphically in a number of ways (e.g., heatmaps and trees) that can facilitate interpretation. We considered trees that are constructed such that individuals with greater genomic similarity are placed next to each other (i.e., they are represented as adjacent branches of the tree) and less similar individuals are represented as branches some distance away from each other, using the module neighbor of the program PHYLIP v. 3.64 (http://evolution.genetics.washington.edu/phylip.html) to construct a neighbor-joining tree. By color coding the individual branches based on the phenotype values possessed by the individuals they represent, one can see if there are patches of a certain color on neighboring branches, which would indicate that phenotype values cluster along with genetic similarity (e.g., using HyperTree v.1.0.0, http://www.kinase.com/tools/HyperTree.html).

### The CEPH-HGDP Cell Line Panel.

We used genotype data from the publicly available CEPH-HGDP Cell Line Panel [56], which have been investigated recently in numerous studies (e.g., reviewed in [4]). The datasets used here include 377 and 783 autosomal microsatellites typed on 1,040 people from 51 populations distributed worldwide (China and United States Han subjects were pooled). We included the same 1,040 subjects as originally described in Rosenberg et al. [1], with the exception of 16 duplicated or mislabeled samples [5]. In addition, we also used geographic data (i.e., the distance from Addis Ababa to each of the 51 CEPH-HGDP populations), kindly provided by Dr. François Balloux [8], and pairwise *F*_ST_ values [82] between 51 populations based on 783 microsatellites kindly provided by Dr. Noah Rosenberg [83].

### The anthropometric data of Howells.

We used craniometric diversity data (the median across the subjects of each of 45 features for each gender) gathered on 489 males and 459 females from ten populations (nine populations for females) made available through the work by Howells [54,55]. The craniometric data was paired according to geographic regions with genetic data from 415 subjects from 19 populations from the CEPH-HGDP panel genotyped on 783 markers as described in Table 1 of Roseman [59]. Pairwise *F*_ST_ between the ten populations (merged from an original 19 CEPH-HGDP populations to represent the locations sampled from, for the craniometric data) was calculated according to standard formulae [82] for diploid data using genotypes at 786 microsatellite loci from the CEPH-HGDP. The pairwise *F*_ST_ analysis produced a 10 × 10 genetic distance matrix that we used in the proposed GAMOVA procedure to determine if relationships exist between the 45 cranial measurements and genetic background similarity.

### The HapMap data set.

We downloaded the ∼700,000 SNP markers from the phase I data available on the 209 individuals genotyped as part of the International HapMap Project (http://www.hapmap.org; [60]). These 209 individuals included 60 individuals of Northern European descent (i.e., the “CEPH-HGDP” derived individuals), 44 individuals of Japanese descent, 45 individuals of Han Chinese descent, and 60 individuals of West African descent (i.e., the “Yoruban” population-derived individuals). Since these 209 individuals had been phased (i.e., assigned haplotypes), we considered the data as providing both 209 multilocus genotypes on each of the 22 autosomes, as well as providing 418 individual chromosomes, from each of the four populations, and analyzed it in this light.

### Power estimations.

A Python computer program was used to generate four sets of two populations, each with *M* markers and *N* subjects with the same constant data size (*D = N* × *M =* 2^20^) as discussed by Patterson et al. [64]. Allele frequencies of all biallelic loci for the first population were generated by assuming they followed a beta-distribution with parameters 0.75 and 0.75. For the second population, for each locus, the allele frequencies of the first population were modified by adding random numbers so that the two populations would exhibit certain genetic distances based on Wright's *F*_ST_ measure of population differentiation ([82], Formula 5.12). For each of the four sets, 1,000 populations were simulated with *F*_ST_ values that ultimately were randomly distributed between 0 and 0.01. We assigned hypothetical individuals in the simulated samples alleles at each of the *M* loci based on the allele frequencies. A GAMOVA analysis was then performed on an IBS distance matrix constructed from the allelic profiles of the simulated individuals as described above with known population membership taken as a predictor variable. Permutations (1,000) of the data were performed to determine the significance of each pseudo-*F* statistic from the GAMOVA analysis.

## Abbreviations

AIM: ancestry informative marker
AMOVA: analysis of molecular variance
ANOVA: analysis of variance
CEPH: Foundation Jean Dausset-Centre d'Etude du Polymorphisme Humain
FRS: nasion-bregma subtense
GAMOVA: generalized analysis of molecular variance
HGDP: CEPH Human Genome Diversity Program
IBS: identical by state
LR: Lynch-Ritland
SNPs: single nucleotide polymorphism
